# Correlation of Serum CA242, CA724, and TPA Levels with Clinicopathological Features and Prognosis in Patients with Inflammatory Bowel Disease Complicated with Rectal Cancer

**DOI:** 10.1155/2022/7742760

**Published:** 2022-09-19

**Authors:** Hongliang Liang, Xi Yang

**Affiliations:** ^1^Department of Gastroenterology, 363 Hospital, Chengdu, 610000 Sichuan, China; ^2^Department of Gastroenterology, The 8th People's Hospital of Chengdu City, Chengdu, 610000 Sichuan, China

## Abstract

**Objective:**

To investigate the correlation of serum cancer antigen 242 (CA242), cancer antigen 72-4 (CA724), and tissue polypeptide antigen (TPA) levels with clinicopathological features and prognosis in patients with inflammatory bowel disease (IBD) complicated with rectal cancer.

**Methods:**

The data of 120 patients with IBD were retrospectively analyzed. Patients were divided into the IBD group (without rectal cancer, *n* = 60) and the rectal cancer group (with rectal cancer, *n* = 60), and 60 healthy individuals receiving medical examination during the same period were selected as the healthy group. Serum CA242, CA724, and TPA levels of research subjects were measured by enzyme-linked immunosorbent assay (ELISA). Meanwhile, the clinical data of the patients were collected. The patients were followed up for 3 years and divided into the survival group and the dead group. The relationship between the levels of CA242, CA724, TPA, and prognosis was tested.

**Results:**

Significant differences were found in the serum CA242, CA724, and TPA levels among three groups (*P* < 0.001). CA242 was related to tumor size, histological stage, growth mode, and TNM stage in patients with IBD and rectal cancer. CA724 was related to histological stage, growth mode, depth of tumor invasion (T stage), lymph node metastasis (N stage), distant metastasis (M stage), and TNM stage in patients with IBD and rectal cancer. TPA was related to histological stage, T stage, M stage, and TNM stage in patients with IBD and rectal cancer. Serum CA242, CA724, and TPA levels in the survival group were significantly lower than those in the dead group after 3 years (*P* < 0.001). As for the combined prediction of serum CA242, CA724, and TPA for patients' prognosis, the confidence interval was 0.000-1.000, AUC was 0.875, standard error was 0.093, and sensitivity was 0.750.

**Conclusion:**

Serum CA242, CA724, and TPA levels are closely related to the clinicopathological features such as location, stage, and metastasis of rectal cancer. The combined detection of serum CA242, CA724, and TPA levels has a significant correlation with the prognosis of patients with rectal cancer, which can be used in monitoring the disease progression.

## 1. Introduction

Inflammatory bowel disease (IBD) is an idiopathic intestinal inflammatory disease involving the ileum and colon, which is difficult to be cured and easy to induce tumor. IBD mainly includes ulcerative colitis and Crohn's disease, with the pathogenic factors of environment, heredity, intestinal microorganism, immune dysfunction, etc. [1, 2], which affects many races around the world. At present, the incidence of IBD in China is on the rise, and more and more patients are affected by chronic mucosal inflammation repeatedly. IBD is complicated with a variety of tumors in the late stage, among which rectal cancer is the most serious one [3, 4]. Compared with sporadic rectal cancer, rectal cancer derived from IBD is more malignant with poor prognosis, and the 5-year survival rate is between 65.2% and 75% [5, 6]. Therefore, the screening of IBD complicated with rectal cancer is of great importance. Endoscopy and pathological examination are the routine clinical diagnosis methods for IBD and rectal cancer. However, invasive examination exerts negative impacts on patients physically and mentally, which is less acceptable [7–9]. Compared with conventional methods, serological examination is more convenient and efficient. Previous studies have shown that CA242 and CA724 are closely related to the degree of tumor differentiation and lymph node metastasis in patients with colorectal cancer [10], and TPA is one of the earliest tumor markers used in the diagnosis of colorectal cancer [11], therefore, they can predict the prognosis of patients with colorectal cancer. In recent years, studies have found that CA242, CA724, and TPA are also abnormally expressed in nonneoplastic diseases, but there are few studies on the expression of CA242, CA724, and TPA in patients with IBD complicated with rectal cancer.

There is a special correlation between IBD and rectal cancer, but little clinical literature combines the two in research, and CA242, CA724, and TPA are often used in the diagnosis of colorectal cancer indistinguishably. Therefore, this paper explored the correlation between serum CA242, CA724, and TPA levels and clinicopathological features and prognosis in patients with IBD complicated with rectal cancer.

## 2. Methods

### 2.1. Patients and Grouping

This study is a retrospective study. A total of 120 patients who were diagnosed and treated in The 8^th^ People's Hospital of Chengdu City from February 2019 to February 2020 were selected as the research objects and divided into the IBD group (without rectal cancer, *n* = 60) and the rectal cancer group (with rectal cancer, *n* = 60). Sixty healthy individuals receiving medical examination were selected as healthy group. Patients in IBD group were 35-75 years old, average age (52.35 ± 2.65), 35 males, and 25 females. Patients in the rectal cancer group were 32-75 years old, average age (52.98 ± 2.45), 36 males, and 24 females. There were 35 males and 25 females in the healthy group, with an average age of 53.10 ± 2.45. This study obtained approval from the Ethics Committee of The 8th People's Hospital of Chengdu City (approval no. 20181164), following the principle of the Declaration of Helsinki (as revised in 2013) [12]. The patients have signed informed consent.

### 2.2. Selection Criteria

The inclusion criteria of IBD group patients were as follows: (1) patients signed the consent form. (2) Patients met the diagnostic criteria in Suggestions on Diagnosis and Treatment of Inflammatory Bowel Disease [13] and presented with different degrees of nausea, abdominal pain, and other symptoms. (3) Patients had no severe complications such as colon cancer and rectal cancer. There were 35 patients with Crohn's disease, 20 patients with ulcerative colitis, and 5 patients with indeterminate colitis, with an average course of disease of (6.21 ± 1.23) months.

The inclusion criteria of rectal cancer group patients were as follows: (1) patients signed the consent form. (2) Patients were diagnosed with rectal cancer by preoperative biopsy or postoperative pathological examination. There were 24 cases of poorly differentiated carcinoma and 36 cases of well differentiated carcinoma as shown in Table 1.

The inclusion criteria of healthy group patients were as follows: (1) individuals signed the consent form. (2) Individuals were matched with the age, gender, and occupation of patients in the IBD group and the rectal cancer group. Exclusion criteria of healthy group patients were as follows: (1) subjects had mental problems or could not be communicated with. (2) Complete clinical data could not be obtained for any reason. (3) Subjects were complicated with liver and kidney dysfunction, severe cardiovascular, and cerebrovascular diseases and multiple primary tumors. (4) Subjects had poor compliance or refused to participate in the research.

### 2.3. Detection of Serum Tumor Markers

8 mL of fasting venous blood in the morning was collected from patients in these three groups and placed in a test tube with separating gel, centrifuged at 3000 r/min for 10 min, and collected patient serum. CA242, CA724, and TPA levels were measured by enzyme-linked immunosorbent assay (ELISA) (Kit: Beijing Kewei Clinical Diagnostic Reagent Co., Ltd., NMPA approval no. S20060028). 96-well ELISA microplates were coated overnight with 100 ml of CA242, CA724, and TPA antibodies at a final concentration of 0.25 mg/L in phosphate-buffered saline (PBS). After washing with 0.05% (*w*/*v*) Tween-20 in PBS (PBST, pH 7.4), the wells were blocked with blocking buffer at room temperature for 1 h. Then, 100 *μ*L of diluted serum samples (1 : 5 dilutions) was added and incubated at room temperature for 2 h. Similarly, 100 *μ*L of PBST lacking antibody was used as a negative control. Following three washes with PBST, 100 *μ*L of antibody diluted to a concentration of 0.25 mg/L was added. After incubation at room temperature for 2 h, 100 *μ*L of avidin-horseradish peroxidase-conjugated secondary antibody (1 : 2000 dilutions) was added, and plates were incubated at room temperature for 30 min. The excess conjugate was removed by washing the plates three times with PBST. The amount of bound conjugate was determined by adding ABTS liquid substrate solution to each well, and plates were incubated at room temperature for color development. The absorbance was measured at 405 nm using a Model 680 microplate reader (Bio-Rad Lab. Inc., Hercules, CA, USA). All analyses were performed in triplicate. The coefficient of variation was lower than 15% between analyses. The positive criteria conformed to the kit, i.e., the normal range of CA242 was 0-12 U/ml, CA724 was 0-6 U/ml, and TPA was 0-125 U/L.

### 2.4. Observation Criteria

1. Serum CA242, CA724, and TPA levels of research subjects were compared
2. The relationship between serum tumor marker levels and clinicopathological features of patients with IBD and rectal cancer. The general data form was fulfilled to analyze the relationship between clinicopathological features and serum CA242, CA724, and TPA levels. The form included patients' name, gender, age, the degree of tumor differentiation, lymph node invasion, tumor size, liver metastasis, depth of tumor invasion (T stage), lymph node metastasis (N stage), distant metastasis (M stage), histological grade, Dukes stage, perineural invasion, and vascular invasion
3. The relationship between serum tumor marker levels and prognosis of patients with IBD and rectal cancer. (1) Patients were followed up for 3 years, and the survival and the dead were recorded. Then, patients were divided into the survival group and the death group, and serum of CA242, CA724, and TPA levels were measured by ELISA. (2) Serum CA242, CA724, and TPA levels were analyzed by receiver operating curve (ROC) to judge the prognostic value. If any one of the levels was positive, the result of combined detection was considered positive

### 2.5. Statistical Analyses

Data were tested by SPSS 20.0. All data here reported are expressed as mean ± SD. *χ*^2^ test was used for intergroup comparison. Independent sample *t*-test was used for comparison between groups. The receiver operating characteristic (ROC) curve was made using Medcalc software. *P* < 0.05 was considerate as statistically significant.

## 3. Results

### 3.1. Comparison of Serum CA242, CA724, and TPA Levels

**Figure 1:**
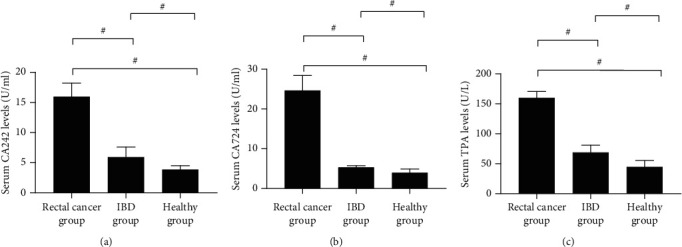
Comparison of serum CA242, CA724, and TPA levels. (a) The comparison of serum CA242 levels. (b) The comparison of serum CA724 levels. (c) The comparison of serum TAP levels. # indicated *P* < 0.001.

The levels of CA242, CA724, and TPA in the healthy group were significantly lower than those in the rectal cancer group and the IBD group (*P* < 0.001), and those in the IBD group were remarkably lower than the rectal cancer group (*P* < 0.001), see Figure 1.

### 3.2. The Relationship between Serum Tumor Marker Levels and Clinicopathological Features of Patients with IBD and Rectal Cancer

CA242 was related to tumor size, histological stage, growth mode, and TNM stage in patients with IBD complicated with rectal cancer. CA724 was related to histological stage, growth mode, T stage, N stage, M stage, and TNM stage in patients with IBD and rectal cancer. TPA was related to histological stage, T stage, M stage, and TNM stage in patients with IBD complicated with rectal cancer (Table 1).

### 3.3. The Relationship between Serum Tumor Marker Levels and Prognosis of Patients with IBD and Rectal Cancer

**Figure 2:**
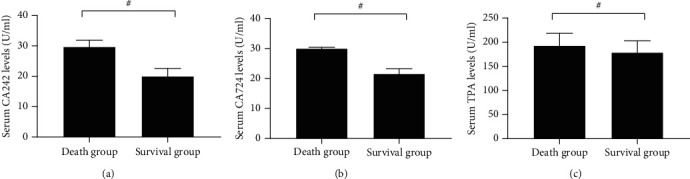
Comparison of serum CA242, CA724, and TPA levels. (a) The comparison of serum CA242 levels. (b) The comparison of serum CA724 levels. (c) The comparison of serum TAP levels. # indicated *P* < 0.001.

**Figure 3:**
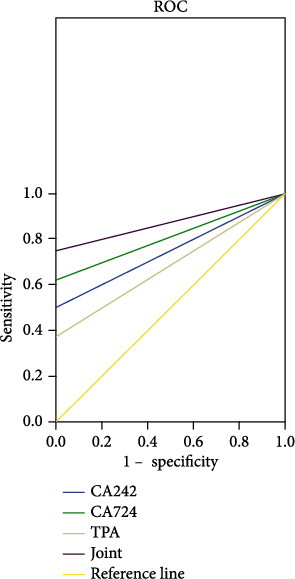
ROC analysis of serum CA242, CA724, and TPA for prognosis prediction.

After 3 years, serum CA242, CA724, and TPA levels in the survival group were significantly lower than those in the death group (*P* < 0.001). The combination of serum CA242, CA724, and TPA predicted patients' prognosis as a confidence interval of 0.000-1.000, AUC of 0.875, standard error of 0.093, and sensitivity of 0.750 (Figures 2 and 3).

## 4. Discussion

IBD is an idiopathic intestinal inflammatory disease involving the ileum, rectum, and colon. Its clinical manifestations include abdominal pain, diarrhea, and bloody stool. Its etiology and pathogenesis are not completely clear. At present, it is generally believed that it is caused by the interaction of multiple factors, mainly including genetic, environmental, and immune factors [14, 15]. As IBD is an independent factor inducing rectal cancer, there is a positive correlation between the incidence and mortality of IBD and rectal cancer [16]. In recent years, with the increase of IBD, the number of patients with rectal cancer also increases every year [17]. According to China's cancer statistics in 2015, the incidence of rectal cancer ranks the fifth among all malignant tumors in China, and the number of patients dying from it is more than 180 thousand every year [18], among which the mortality rate of rectal cancer triggered by IBD is significantly higher than that of diffuse rectal cancer, with lower 5-year survival rate of patients and the poor prognosis [19]. The reported risk of rectal cancer in patients with a retained rectum following surgery for IBD varies considerably in the literature, with a reported incidence as low as 0% and as high as 12% [20]. With the progress of medical technology, the diagnostic rate of IBD and rectal cancer continues to improve, but they all depend on the comprehensive clinical manifestations, endoscopic manifestations, imaging, and pathological results. After excluding other intestinal diseases, the diagnosis is established, and there is a lack of rapid, simple, and noninvasive indicators for differential diagnosis.

Improving disease surveillance is the crux to reduce the mortality of patients with IBD complicated with rectal cancer. At present, proteomics, fecal DNA, and endoscopy are mainly used to screen rectal cancer in practice. However, each method has certain disadvantages, such as poor patient compliance, which is not conducive to long-term monitoring. The detection of serum tumor markers is a widely used examination method in recent years, which can be performed repeatedly, and reduce the physical and mental pressure of patients and their medical burden. A number of studies have shown that CA242, CA724, and TPA are expressed in a variety of tumors and related diseases, but there are few studies on the difference of their expression levels in patients with inflammatory bowel disease and rectal cancer [21]. The serum CA242, CA724, and TPA selected in this study are closely related to the progression of IBD and rectal cancer. Under healthy conditions, the CA242, CA724, and TPA synthesized by the body exist in the cells. With the development of the disease to malignant, the cell structure of the patients will be destroyed. The more serious the destruction is, the more CA242, CA724, and TPA enter the blood circulation [22]. IBD, as a precancerous lesion of rectal cancer, has a significantly lower malignant degree. Therefore, there are statistical differences in serum CA242, CA724, and TPA levels among healthy group, IBD group, and rectal cancer group. The diagnostic thresholds of CA242, CA724, and TPA are the highest in rectal cancer. Therefore, the combination of the three can be used to distinguish IBD and rectal cancer and provide basis for clinical monitoring of disease progress.

The application of CA242, CA724, and TPA in the detection of colorectal cancer has been difficult to distinguish [23], and the efficacy of the three in rectal cancer has not been studied, especially the relationship between CA242, CA724, and TPA and rectal cancer caused by IBD. CA242 is a mucin glycoantigens, with a lower level in patients with benign diseases but a higher level in patients with gastrointestinal malignant diseases, which has the high sensitivity to colorectal cancer, and its diagnostic efficiency is better than that of general tumor markers. Bezzio et al. believed that CA242 was closely related to tumor diameter, and the level of CA242 increased with the increase of tumor diameter [24]. In the study of Nair et al., CA242 level could reflect tumor location and tumor stage [25]. This study also found that CA242 was related to tumor size, histological stage, growth mode, and TNM stage in patients with IBD complicated with rectal cancer. CA724 is carbohydrate antigen, with significantly higher detection rate of colorectal cancer than other markers like carcinoembryonic antigen. CA724 level increases as the invasion expand. In this paper, CA724 was related to histological stage, growth mode, T stage, N stage, M stage, and TNM stage in patients with IBD and rectal cancer, showing that CA724 could be used to judge lymph node metastasis and was one of the best indicators of disease grade and stage. TPA is a mixture of low molecular weight keratins, which produces a large amount of keratins during cell proliferation, and is generally regarded as a sign of cell proliferation in clinic. This index is related to histological stage, T stage, M stage, and TNM stage in patients with IBD complicated with rectal cancer.

The American Cancer Society suggests that tumor markers cannot be used as a diagnostic index alone [26], and serum CA242, CA724, and TPA are closely related to the clinicopathological features of IBD complicated with rectal cancer. The three can jointly predict the prognosis of patients. The serum CA242, CA724, and TPA levels in the survival group were significantly lower than those in the death group after 3 years. The confidence interval of the three factors in predicting the prognosis of patients is 0.000-1.000, the AUC is 0.875, the standard error is 0.093, and the sensitivity is 0.750, indicating that the three factors are highly correlated with the prognosis of patients, which is helpful to correctly evaluate the prognosis. It should be noted that the patients in the groups received different treatment methods in 3 years, which may affect the experimental results to a certain extent, so dynamic monitoring of serum CA242, CA724, and TPA levels is extremely important.

## 5. Conclusion

Serum CA242, CA724, and TPA levels are closely related to the clinicopathological features such as location, stage, and metastasis of rectal cancer. The combined detection of CA242, CA724, and TPA levels has high diagnostic value and can be used as an indicator for early screening of IBD and rectal cancer.

## Figures and Tables

**Table 1:** The relationship between serum tumor marker levels and clinicopathological features in patients with IBD and rectal cancer ($\bar{x}\pm s$).

Clinicopathological features	*n*	CA242 (U/ml)	*t*	*P*	CA724 (U/ml)	*t*	*P*	TPA (U/L)	*t*	*P*
Gender			0.738	0.463		0.607	0.546		1.206	0.233
Male	36	16.20 ± 2.11			24.89 ± 2.14			162.95 ± 10.10		
Female	24	15.78 ± 2.23			24.55 ± 2.10			159.68 ± 10.58		
Age (years old)			1.081	0.284		0.530	0.598		0.910	0.367
≥50 years old	34	16.23 ± 2.10			24.80 ± 2.13			161.55 ± 11.10		
<50 years old	26	15.64 ± 2.09			24.50 ± 2.23			158.78 ± 12.41		
Tumor size (cm)			11.888	<0.001		0.786	0.435		0.577	0.566
≥5	32	18.44 ± 1.98			24.99 ± 2.10			161.25 ± 10.45		
<5	28	12.76 ± 1.68			24.56 ± 2.13			159.68 ± 10.58		
Histological grades			3.146	0.012		6.223	0.012		2.653	0.023
G1	15	13.10 ± 2.11			18.41 ± 2.23			149.23 ± 10.68		
G2	40	15.21 ± 2.13			24.55 ± 2.23			156.98 ± 12.14		
G3	5	17.42 ± 2.10			26.85 ± 2.14			169.79 ± 12.65		
Growth modes			13.029	<0.001		5.157	0.040		2.660	0.096
Ulcerative	30	14.23 ± 1.25			22.14 ± 2.65			156.21 ± 12.58		
Invasive	25	20.12 ± 1.10			26.98 ± 2.14			168.98 ± 12.65		
Contracted	5	11.10 ± 1.54			20.15 ± 2.10			149.65 ± 11.65		
T stages			3.532	0.001		10.081	<0.001		4.392	<0.001
*T*_1−2_	20	14.26 ± 1.25			22.10 ± 2.12			155.26 ± 11.54		
*T*_3−4_	40	15.65 ± 1.52			28.14 ± 2.22			169.23 ± 11.65		
N stages			0.783	0.437		8.461	<0.001		0.955	0.344
*N*_0_	32	5.89 ± 1.23			10.63 ± 2.11			159.65 ± 12.10		
*N*_1−2_	28	15.68 ± 1.44			21.95 ± 2.13			162.58 ± 11.58		
M stages			1.208	0.232		8.866	<0.001		3.048	0.004
*M*_0_	52	15.55 ± 1.23			21.23 ± 2.20			154.98 ± 12.50		
*M*_1_	8	16.23 ± 2.68			28.65 ± 2.23			169.21 ± 10.65		
TNM stages			3.901	<0.001		9.669	<0.001			<0.001
I-II	30	14.59 ± 1.26			22.65 ± 2.14			153.65 ± 12.10		
III-IV	30	16.68 ± 2.65			27.98 ± 2.13			166.54 ± 11.58		
Perineural invasion			0.153	0.879		0.687	0.495		0.601	0.550
*Y*	5	15.85 ± 2.12			24.30 ± 2.12			158.65 ± 12.65		
*N*	55	16.00 ± 2.10			24.98 ± 2.12			162.20 ± 12.65		
Vascular invasion			0.287	0.775		1.426	0.159		0.477	0.656
*Y*	6	15.75 ± 2.16			24.23 ± 1.59			159.65 ± 13.26		
*N*	54	16.01 ± 2.10			24.99 ± 1.20			162.20 ± 13.25		

## Data Availability

The datasets during the current study are available from the corresponding author on reasonable request.
